# After chemo-metamorphosis: *p*-menthane monoterpenoids characterize the oil gland secretion of adults of the oribatid mite, *Nothrus palustris*

**DOI:** 10.1007/s00049-023-00386-y

**Published:** 2023-07-20

**Authors:** Günther Raspotnig, Michaela Bodner, David Fröhlich, Julia Blesl, Edith Stabentheiner, Olaf Kunert

**Affiliations:** 1 Institute of Biology, University of Graz, Graz, Austria; 2 Institute of Pharmaceutical Sciences, University of Graz, Graz, Austria

**Keywords:** Opisthonotal glands, Astigmatid compounds, Dehydrocineole, Geranial, Chemosystematics, *p*-1,8-Menthadien-5-ol

## Abstract

The oil gland secretion of the oribatid mite *Nothrus palustris* is known to show the phenomenon of juvenile–adult polymorphism, i.e., juvenile instars produce secretions predominated by geranial, whereas adults secrete dehydrocineole along with a number of chemically unidentified compounds. We here re-analyzed the secretions of adult *N. palustris* by GC–MS and NMR spectroscopy, eventually identifying the unknown compounds as *p*-menthane monoterpenoids. The major components were two isomeric 6-isopropenyl-3-methyl-cyclohex-3-en-1-yl formates (= *p*-1,8-menthadien-5-yl formates), which accounted for about 75% of the secretion. These were accompanied by five additional, only partly identified *p*-menthanes (or *p*-methane-derivatives), all of which represented minor or trace components. In addition, adult secretions contained two C_21_-hydrocarbons, 1,12-heneicosadiene (major) and a heneicosatriene (minor). Menthane monoterpenoids represent a novel sub-class of terpene compounds in the oil gland secretions of Oribatida. In case of *N. palustris*, we assume that both geranial and *p*-menthane monoterpenoids arise via the mevalonate pathway which obviously shows a split at the level of geranyl pyrophosphate, leading to geranial in juveniles and to *p*-menthanes in adults. The significance of methane occurrence in oil glands as well as the taxonomic distribution of juvenile–adult polymorphism in oribatid oil gland secretions is discussed. The latter phenomenon—i.e., “chemo-metamorphosis” of secretions—is not known from early- and middle-derivative Oribatida nor from Astigmata, but appears to be more common in some derivative desmonomatan and brachypyline oribatid groups.

## Introduction

Oil glands constitute paired opisthosomal exocrine glands, that characterize the majority of Oribatida (“glandulate Oribatida”) and the Astigmata (Norton 1998). If present in a species, oil glands occur in all ontogenetic stages, from larvae to adult individuals (Van der Hammen 1980), representing sources of various pheromones, repellents, and antimicrobial compounds (Shimano et al. 2002; Raspotnig 2006; Kuwahara 1991, 2004; Heethoff et al. 2011a).

The secretions of oil glands have mainly been studied in early- and middle-derivative Oribatida (e.g., Sakata and Norton 2001, 2003; Raspotnig et al. 2001, 2004, 2005a, b, 2008, 2009; Heethoff et al. 2018) and, even more extensively, in Astigmata (e.g., Kuwahara et al. 1975; Kuwahara 2004). A major chemical trait in these groups is a combination of specific hydrocarbons, terpenes, and aromatics which are arranged in species-specific chemical profiles (Sakata and Norton 2001; Raspotnig et al. 2001). Such profiles do not appear to change dramatically during ontogenetic development, so that all stages of a particular species—from larvae up to adult individuals—show the same oil gland chemistry. In *Collohmannia gigantea* Sellnick 1922, for instance, adults produce a blend of 2-hydroxy-6-methyl benzaldehyde, neral, geranial, neryl formate, γ-acaridial, tri- and pentadecane, which is very similar in juveniles (Raspotnig et al. 2001; Raspotnig 2006). A lack of juvenile–adult polymorphism is also found in the desmonomatans *Archegozetes longisetosus* Aoki, 1965 (Sakata and Norton 2003)*, Platynothus peltifer* (C.L. Koch 1839) (Raspotnig et al. 2005b) as well as in different species of *Trhypochthonius* Berlese 1904 (Raspotnig et al. 2004; Heethoff et al. 2011b). To the best of our knowledge, there is no evidence of altered compositions of secretions during ontogeny in the Astigmata either (Kuwahara 1991, 2004).

About 20 years ago, a report on a first incidence of juvenile–adult polymorphism of oil gland secretions in Oribatida was published: When investigating the oil glands of the desmonomatan *Nothrus palustris* (C.L. Koch 1839), Shimano et al. (2002) found that juvenile stages—but not adults—produced an alarm pheromone, geranial. Adult secretions, by contrast, contained a rich set of novel compounds, of which only two, namely dehydrocineole and a C_21_-hydrocarbon (heneicosadiene), were identified.

We here re-analyzed the secretion of *N. palustris* with the aim to identify the remaining compounds. We eventually show that it is a blend of *p*-menthane monoterpenoids that replace juvenile geranial and predominate in the secretions of *N. palustris* after the final moult.

## Materials and methods

About 215 adult individuals and 14 juveniles of *N. palustris* were extracted (by Berlese–Tullgren extraction) from sieved soil samples collected during the years 2022/2023 at different locations in Styria, Austria, namely (1) near the “Fischteich” in Passail, Styria, Austria (N 47.2755; E 15.5326); (2) in Pirching am Traubenberg (N 46.9330; E 15.6111), and (3) in Heiligenkreuz am Waasen (N 46.9644; E 15.5829). Forty adult individuals from different populations were used to prepare individual whole-body extracts (single individuals were extracted in 20 µl methylene chloride for 15 min). Crude extracts, containing extruded oil gland secretion, were used for gas chromatography–mass spectrometry (GC–MS). To determine the double-bond position of unsaturated hydrocarbons, 16 individuals were extracted in 100 µl hexane for 15 min. The remaining adult individuals (about 160) were used to prepare a pooled extract in 720 µl deuterated chloroform (CDCl_3_) for nuclear magnetic resonance spectroscopy (NMR). Additionally, ten extracts from juvenile stages were prepared: one pooled larval extract (five larvae in 20 µl methylene chloride) and three individual extracts from each nymphal stage (proto-, deuto-, and tritonymphs; each individual in 20 µl methylene chloride).

Instrumentation/conditions for GC–MS: We measured on a Trace GC-DSQI instrument from Thermo (Vienna, Austria). The GC was equipped with a ZB-5 capillary column (30 m × 0.25 mm × 0.25 µm) which was heated as follows: 50 °C for 1 min, then increase by 10 °C/min to 300 °C, and a 5 min isothermal hold. Helium (at a constant flow rate of 1.2 ml/min) was the carrier gas. The injector was kept at 240 °C; the transfer line at 310 °C. The MS worked in electron impact (EI) mode at 70 eV. The ion source was at 200 °C; we scanned ions from mass/charge ratio 40–500.

Some measurements (particularly those for the localization of double bonds, see text) were done on a 5977B GC/MSD (coupled to an 8890 GC) from Agilent (Vienna, Austria). We used two series connected HP-5MS ultra inert capillary columns, each 15 m × 0.25 mm × 0.25 µm, at helium flow rates of 1.0 and 1.2 ml/min, respectively, and the same MS parameters as listed above. For the detection of the DMDS derivatives, we relied on a slightly longer temperature program, starting at 40 °C, ramping by 10 °C/min to 300 °C, and keeping 300 °C for 15 min.

Instrumentation/conditions for NMR: NMR experiments (1D ^1^H, and 2D COSY, HSQC, HMBC, and HSQC-TOCS) were performed with a Bruker 700 MHZ Avance II NMR spectrometer (Rheinstetten, Germany) equipped with a cryo-probe. For the HSQC, a version with multiplicity editing was used.

Data evaluation/reference compounds: GC–MS data were evaluated with XCalibur 2.07 (Thermo) and MassHunter Workstation 10.0 (Agilent). For a first approach to identify compounds, we used the NIST05 mass spectrometric library.

To fully identify compound A (dehydrocineole), 2,3-dehydro-1,8-cineole was synthesized according to already published procedures (Carman et al. 2005; Brenna et al. 2013). For compounds B and C, GC–MS data in combination with NMR data were used for identification. The hydrocarbons D and VI were identified by their mass spectra, and the positions of double bonds in compound D were determined by DMDS derivatization (Carlson et al. 1989; Fröhlich et al. 2022). The remaining minor compounds (I–V) were tentatively identified by GC–MS data only. Normal alkane retention indices (Van den Dool and Kratz 1963) were calculated using an alkane-standard (C8-C40). Relevant compounds for the synthesis of dehydrocineole [eucalyptol, N-bromo succinimide, dibenzoyl peroxide, azobis(isobutyronitrile)] as well as the alkane standard were purchased from Sigma (Vienna, Austria); tetrahydrofuran, dimethylformamide, and dimethylsulfoxide were from Roth (Graz, Austria); α-terpineol was from TCI Europe (Eschborn, Germany).

To visualize position and morphology of oil gland pores, scanning electron micrographs (SEM) were taken as follows: a clean, adult individual of *N. palustris* was fixed in ethanol, dehydrated in 100% ethanol and acetone, air dried overnight, then mounted onto Aluminium pin stubs (Agar Scientific, Biedermannsdorf, Austria), sputtercoated with gold (Agar Sputter Coater; Christine Gröpl, Tulln, Austria), and investigated with a Hitachi FlexSEM1000 (Tokyo, Japan) using 20 kV acceleration voltage in the high-vacuum mode.

## Results

**Fig. 1:**
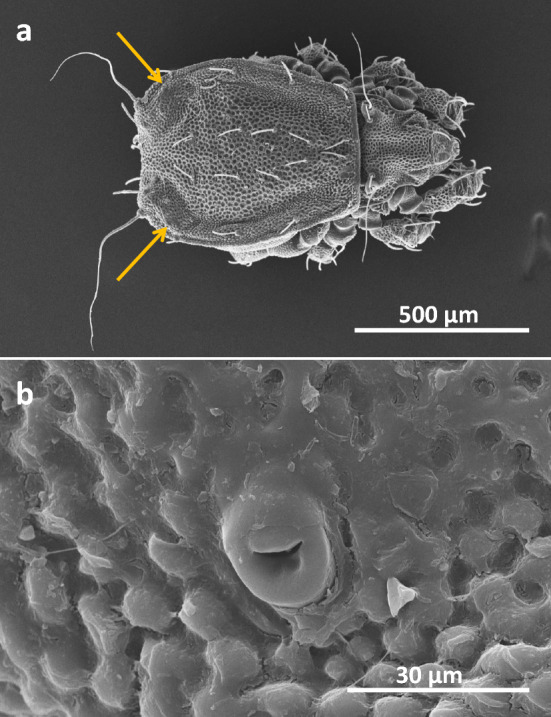
Scanning electron micrographs of an adult individual of *Nothrus palustris* showing the location of oil gland pores (**a**: arrows) and details of right pore (**b**)

Oil glands of *N. palustris* are large paired glands located beneath slight bulges at the distal edges of the notogaster (Fig. 1a). They open dorso-laterally at each edge of the notogaster via a small, mouth-shaped pore surrounded by smooth cuticular lips (pore diameter: about 15 µm) (Fig. 1b).

**Fig. 2:**
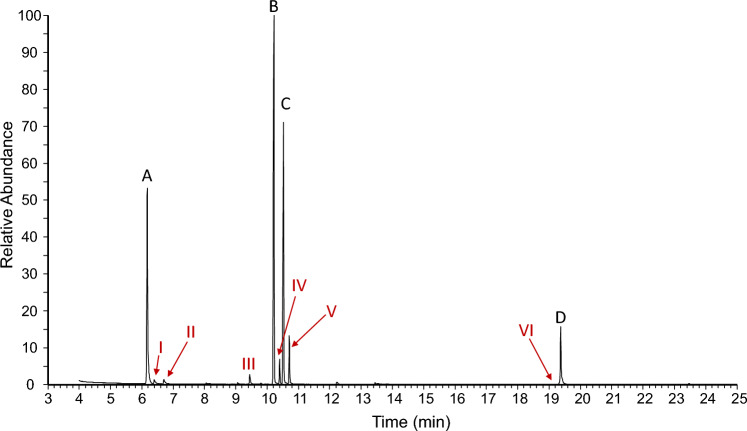
Characteristic chemical profile of the oil gland secretion of adult *Nothrus palustris*. Major compounds (black): peak **A** (2,3-dehydro-1,8-cineole), **B** ((1*R*,6*R*)-or (1*R*,6*R*)-*p*-1,8-methadien-5-yl formate), **C** ((1*R*,6*S*)-or (1*S*,6*R*)-*p*-1,8- methadien-5-yl formate), and **D** (1,12-heneicosadiene). Minor compounds (red): peak I (menthane-hydrocarbon C_10_H_14_, possibly menthatriene isomer 1), peak II (menthane-hydrocarbon C_10_H_14_, possibly menthatriene isomer 2), peak III (mono-oxygenated *p*-menthane C_10_H_16_O, possibly *p*-1,8-menthadien-5-ol), peak IV (mono- or doubly oxygenated *p*-menthane C_12_H_20_O or C_11_H_16_O_2_, isomer 1), peak V ((mono- or doubly oxygenated *p*-menthane C_12_H_20_O or C_11_H_16_O_2_, isomer 2), and peak VI (heneicosatriene)

**Fig. 3:**
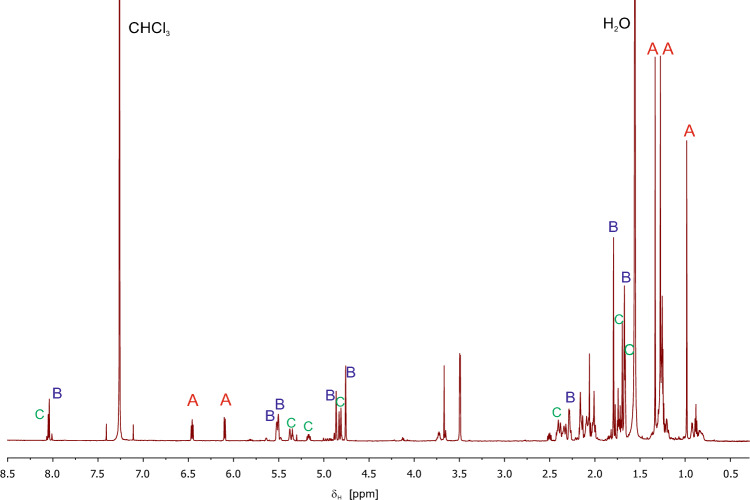
Proton NMR spectrum (700 MHz, CDCl_3_) of the *N. palustris* chloroform extract. Resonances of the three assigned compounds **A**–**C** are labeled accordingly

Whole-body extracts of adult individuals consistently showed four major peaks (A-D), together amounting for about 90% of the extract profile (Fig. 2, Table 1). The proton NMR spectrum (Fig. 3) of the extract (160 adults) was dominated by the three compounds A, B, and C. Their molecular constitutions were determined in mixture by complete assignments of their resonances in the 2D NMR spectra (Table 2).

**Table 1:** Gas chromatographic—mass spectrometric data to extract components of adult *Nothrus palustris*

Peak no	RI*	EI-fragmentation pattern [*m/z* (rel. Int.)]	Rel. abundance**	Identified as
*Major peaks*				
A	995	152(6), 124(21), 109(100), 94(21), 91(9), 79(38), 77(13), 43(13)	24	2,3-Dehydro-1,8-cineole
B	1267	180(< 1), 134(67), 119(100), 117(8), 107(6), 106(13), 105(30), 93(24), 92(31), 91(45), 84(16), 83(29), 79(14), 77(16), 41(11)	34	(4*R*,5*R*)- or (4*S*,5*S*)-*p*-1,8-menthadien-5-yl formate = (1*R*,6*R*)- or (1*S*,6*S*)-6-isopropenyl-3-methyl-cyclohex-3-en-1-yl) formate
C	1288	180(< 1), 134(67), 119(100), 117(8), 107(6), 106(11), 105(25), 93(22), 92(24), 91(41), 84(9), 83(13), 79(14), 77(14), 67(6), 65(5), 55(6), 41(9)	25	(4*R*,5*S*)- or (4*S*,5*R*)-*p*-1,8-menthadien-5-yl formate = (1*R*,6*S*)- or (1*S*,6*R*)-6-isopropenyl-3-methyl-cyclohex-3-en-1-yl) formate
D	2069	292(13), 180(4), 166(7), 152(8), 138(13), 137(11), 124(23), 123(24), 110(35), 109(41), 97(46), 96(85), 95(67), 83(73), 82(100), 81(84), 70(19), 69(75), 68(48), 67(65), 57(28), 56(29), 55(86), 54(33), 43(28), 41(44)	8	1,12-Heneicosadiene (1,12-C_21:2_)
*Minor peaks (tentatively identified)*				
I	1009	134(61), 119(100), 117(17), 115(14), 105(31), 93(21), 92(35), 91(97), 79(14), 77(32), 65(9), 41(8)	< 1	*p*-Menthane monoterpene C_10_H_14_ (isomer1)
II	1029	134(70), 119(72), 117(12), 115(7), 106(14), 105(26), 103(8), 93(14), 92(27), 91(100), 79(23), 77(34), 65(9), 51(6), 41(9)	< 1	*p*-Menthane monoterpene C_10_H_14_ (isomer 2)
III	1213	152(5), 137(100), 134(20), 119(87), 117(9), 109(63), 108(61), 107(35), 106(19), 105(28), 97(25), 95(19), 94(23), 92(25), 91(72), 84(76), 83(70), 82(20), 81(27), 79(44), 77(35), 69(25), 68(22), 67(37), 65(13), 56(15), 55(19), 53(17), 43(11), 41(32)	1	Oxygenated *p*-menthane monoterpenoid C_10_H_16_O (probably *p*-1,8-menthadien-5-ol)
IV	1280	180(< 1), 165(< 1), 152(2), 137(8), 134(100), 119(77), 117(13), 115(9), 112(31), 106(14), 105(26), 94(10), 93(23), 92(39), 91(75), 84(71), 83(48), 79(22), 77(30), 67(19), 65(12), 41(17)	2	Oxygenated *p*-menthane monoterpenoid C_10_H_18_O_2_ (isomer 1)
V	1302	180(< 1), 165(< 1), 152(1), 137(4), 134(100), 121(6), 119(64), 117(10), 115(7), 112(19), 107(11), 106(12), 105(22), 94(20), 93(23), 92(22), 91(63), 84(47), 83(32), 79(30), 77(27), 67(8), 65(10), 55(7), 41(12)	4	Oxygenated *p*-menthane monoterpenoid C_10_H_18_O_2_ (isomer 2)
VI	2069	290(5), 191(2), 163(2), 150(7), 149(6), 138(16), 135(16), 124(12), 123(17), 122(14), 121(22), 110(32), 109(27), 107(13), 96(52), 93(27), 83(21), 82(69), 81(100), 80(57), 79(29), 77(14), 69(25), 68(44), 67(98), 57(13), 55(48), 54(18), 43(10), 41(30)	< 1	Heneicosatriene (C_21:3_)

*normal alkane retention index (Van den Dool and Kratz 1963). **%peak area of total extract components

**Table 2:** NMR spectrometric data (700 MHz, 175 MHz, CDCl_3_) of extract components **A**–**C** of adult *Nothrus palustris* and literature data of the acetate homologue of **C**

Atom	A		B		C		(4*S*, 5*R*) homologue of C (synthetic acetate)	
	δ_C_	δ_H_, (*J* in Hz)	δ_C_	δ_H_, (*J* in Hz)	δ_C_	δ_H_, (*J* in Hz)	δ_C_	δ_H_, (*J* in Hz)
1	70.9	–	129.8	–	130.8	–		
2	135.2	6.10 dd (8.2, 1.0)	120.5	5.52	120.2	5.37 brs	120.6	5.35 t (4.6)
3	135.3	6.46 t (7.3)	25.1	2.34 2.08	30.2	2.16 2.16	30.8	2.10 – 2.15
4	39.4	2.28	42.6	2.28	46.1	2.50	46.8	2.32 – 2.45
5	20.3	2.02 1.20 tdd (12.3, 5.1, 2.3)	69.2	5.50	71.5	5.17	72.0	5.05
6	31.1	1.73 1.26	36.3	2.39 2.15	35.7	2.40 2.08	36.6	2.32 – 2.45
7	24.7	1.33 s	23.1	1.67 s	23.0	1.66 s	23.5	1.65—1.72
8	74.2	–	144.9	–	145.3	–	146.2	–
9	29.1	0.98 s	111.3	4.87 brs 4.76	112.6	4.83 brs 4.81 brs	112.7	4.75 m 4.75 m
10	28.1	1.28 s	22.4	1.79 s	19.3	1.70 s	20.1	1.65—1.72
formate			160.8	8.04 s	160.8	8.05 s		

The EI mass spectrum and the NMR data of peak A (M^+^ at *m/z* 152, base ion at *m/z* 109) indicated 2,3-dehydro-1,8-cineole, as already identified from adult extracts in a previous study (Shimano et al. 2002). The identity of the compound as 2,3-dehydro-1,8-cineole was confirmed by synthesis.

Peaks B and C appeared to be isomeric compounds, exhibiting indistinguishable mass spectra (Table 1). Molecular ions in both components were weak, but could be detected at *m/z* 180 (intensity < 1%). Major fragment ions were observed at *m/z* 134, 119, 105, 91, and 77, corresponding to a *p*-menthane monoterpenoid structure. Hits from NIST05 could not be confirmed. However, the fragment at *m/z* 134 (M^+^—46: C_10_H_14_^+^), probably arising from the neutral loss of formic acid from the molecular ion, tentatively indicated an ester structure. Assignment of the NMR resonances led to two isomeric *p*-*1,8*-menthadien-5-yl formates—comprising a limonene backbone with a formyl group in position 5—with different carbon chemical shifts at the two asymmetric carbons C-4 and C-5 and the methylene group C-3 indicating the presence of isomers or two pairs of enantiomers with different relative configurations in the extract. The NMR resonances with the higher intensities were correlated with compound B (higher peak area in GC) and the NMR resonances with lower intensity, consequently, with compound C. While both compounds have not been described in literature yet, the chemical synthesis of a homologue (4*S*,5*R*)-acetate has already been reported (Brenna et al. 2006). As the carbon resonance values of the synthetic compound (Table 2) are in very good agreement with compound C in the extract, the relative configuration of C has to be (4*S*,5*R*) and the relative configuration of B has to be (4*R*,5*R*). On the basis of the NMR spectra, the compounds B and C determined as (1*R*,6*R*)- or (1*S*,6*S*)-6-isopropenyl-3-methyl-cyclohex-3-en-1-yl formate for compound B [= (4*R*,5*R*)- or (4*S*,5*S*)-*p*-1,8-menthadien-5-yl-formate] and (1*R*,6*S*)- or (1*S*,6*R*)- 6-isopropenyl-3-methyl-cyclohex-3-en-1-yl formate [= (4*S*,5*R*)- or (4*R*,5*S*)-*p*-1,8-menthadien-5-yl-formate] for compound C, respectively.

**Fig. 4:**
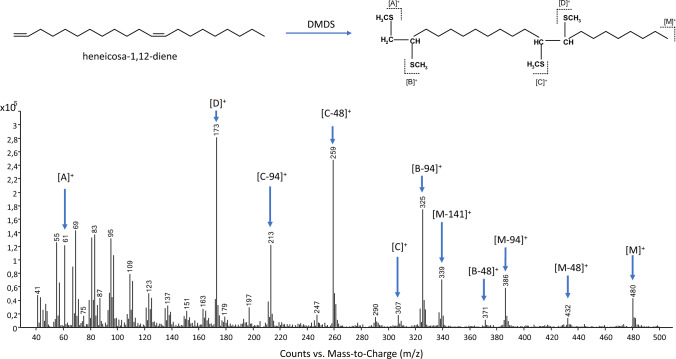
Localization of double bonds in the *Nothrus*-heneicosadiene. Diagnostic ions of the DMDS derivative were interpreted according to Carlson et al. (1989): In a nutshell, DMDS diadducts of alkadienes theoretically show (1) a molecular ion at 188 amu higher than in the original compound (= plus 4 × SCH_3_); (2) fragments arising from the loss of HSCH_3_ (− 48 amu), CH_3_SSCH_3_ (− 94 amu), and SCH_3_ + CH_3_SSCH_3_ (− 141 amu) from the molecular ion; (2) two complementary pairs of fragments ([A]^+^ and [B]^+^, [C]^+^ and [D]^+^), arising from cleavage of C–C bonds between the SCH_3_-substituents (= at the sites of double bonds in the original molecule, allowing to locate the regarding positions); and 4) loss of HSCH_3_ (- 48 amu) and CH_3_SSCH_3_ (- 94 amu) from fragments ([B]^+^ and [C]^+^) that bear three SCH_3_-groups

Compound D was identified as a C_21_-alkadiene, mainly by interpretation of its mass spectrum (M^+^ at *m/z* 292; Table 1). The positions of the double bonds were determined by DMDS derivatization (Table 3, Fig. 4). Initially, a 6,9-heneicosadiene—as proposed by Shimano et al. (2002)—was expected. Diadducts of alkadienes with double bonds separated by only one CH_2_ unit (as in 6,9-heneicosadiene) show the formation of cyclic thioethers, along with a loss of 62 amu (CH_3_SCH_3_) by adduct formation, as described in detail in Raspotnig et al. (2005b). In the present case, such a loss (theoretically leading to a fragment at *m/z* 418: 292 + 4 × SCH_3_—62) was not observed, indicating that the two double bonds were not adjacent.

**Table 3:** Localization of double-bond position: diagnostic ions in the DMDS derivative of heneicosadiene (peak D)

	[A]^+^	[D]^+^	[B—94]^+^	[B—48]^+^	[B]^+^	[C—94]^+^	[C—48]^+^	[C]^+^	[M—141]^+^	[M—94]^+^	[M—48]^+^	[M]^+^
*m/z*	61	173	325	371	–	213	259	307	339	386	432	480

Diagnostic fragments were interpreted following Carlson et al. (1988), as outlined in detail in the text and shown in Fig. 4. Fragment ion [B]^+^, probably instable by containing three SCH_3_ groups and theoretically at *m/z* 419, was not detected

We indeed observed a molecular ion an *m/z* 480 (= 292 plus 4 × SCH_3_) along with characteristic losses of SCH_3_-groups from M^+^ at *m/z* 432 (M^+^—48), *m/z* 386 (M^+^—94) and *m/z* 339 (M^+^—141). The fragments from cleavage of C–C bonds between the outer SCH_3_-bearing groups were found at *m/z* 61 (= [A]^+^) and 173 (= [D]^+^), indicating one double bond in position 1 (61—SCH3 = 14; i.e., only one CH_2_ unit in this fragment), the other in position 9 from the other end of the molecule (173—SCH3 = 126: i.e., 9 CH_2_-units in this fragment). Position 9 from the other end of a heineicosadiene corresponds to position 12 in regular direction (21Cs in total minus a 9 C-fragment = position 12). The remaining two fragments from cleavage of C–C bonds between the inner SCH_3_-bearing groups bear three SCH_3_-groups (= [B]^+^ and [C]^+^) and represent the complementary fragments to [A]^+^ and [D]^+^. Thus, fragment [A]^+^ plus [B]^+^ should give the whole molecule (= M^+^ at *m/z* 480), and [D] + and [C] + should also give 480. Fragment [B]^+^ was expected at *m/z* 419 (480—61), and [C]^+^ at *m/z* 307 (480–173). Such fragments generally show further losses of 48 and 94 mass units (= one and two SCH_3_-groups, respectively), facilitating their detection. In our case, fragment [B]^+^ itself was not observed, but ions [B—48]^+^ (at *m/z* 371) and [B—94]^+^ (at *m/z* 325) were visible. On the other hand, fragment [C]^+^ was observed at *m/z* 307, along with [C—48]^+^ (at *m/z* 259) and [C—94]^+^ (at *m/z* 213). This data together clearly indicated a doubly unsaturated C_21_-alkadiene with double bonds in positions 1 and 12 (i.e., a 1,12-heneicosadiene) (Fig. 4, Table 3).

A further (but minor) hydrocarbon, obviously a triple-unsaturated analog (peak IV: M^+^ at *m/z* 290, heneicosatriene), accompanied 1,12-heneicosadiene. Due to the low quantity of the compound, it could not be recovered after derivatization.

Adult extracts exhibited a number of additional, minor peaks, all of which showed mass spectra of *p*-menthanes or menthane derivatives. All these minor compounds together amounted for less than 10% of the total peak area of the extracts. Five consistently occurring minor components (peaks I–V) were further investigated (Table 1), though straightforward identifications via MS were hampered by weak molecular ions. Peaks I and II, however, showed intense molecular ions *m/z* at 134 and fragments at *m/z* 119 and 91. A library search pointed to compounds, such as dimethyl-octatetraenes, cymenes, and menthatrienes. For well-documented *p*-menthatrienes (such as 1,3,8- and 1,5,8-*p*-methatriene) and 2,6-dimethyl-1,3,5,7-octatetraene, the observed retention indices were too low (see discussion). For *p*-, *o*-, and *m*-cymenes, reported mass spectra and indices fairly fitted (e.g., Adams et al. 2012), but a direct comparison to authentic standards did not show full correspondence. However, based on the prevalence of *p*-menthanes in the secretion (e.g., peaks B, C, III), we tentatively assume compounds I and II to be isomeric menthatrienes.

Peak III appeared to be a mono-oxygenated *p*-menthane monoterpenoid of formula C_10_H_16_O, exhibiting a visible molecular ion at *m/z* 152, a base ion at *m/z* 137 (M^+^-CH_3_), as well as elimination of water from M^+^ (*m/z* 134). We assume that peak III represented the corresponding alcohol to the *p*-menthane formates B and C, i.e., 6-isopropenyl-3-methyl-cyclohex-3-en-1-ol (= *p*-1,8-menthadien-5-ol = limonen-5-ol).

Peaks IV and V also showed the mass spectra of isomeric *p*-menthane monoterpenoids. The molecular ions were weak, and well-visible fragments of highest mass were recorded at *m/z* 137, with a base ion at *m/z* 134. We thus expected a molecular ion at *m/z* 152 (again indicating a menthadienol), but then found weak fragments at *m/z* 180 and 165. Proposing a molecular mass of 180 amu, the molecules theoretically contained one or two oxygens and thus corresponded to a molecular formula of either C_12_H_20_O or C_11_H_16_O_2_, respectively.

By contrast, extracts of all juvenile stages (i.e., larvae, proto-, deuto-, and tritonymphs) were predominated by one single peak, geranial (M^+^ at *m/z* 152), as already reported by Shimano et al. (2002).

## Discussion

### p-Mentha-1,8-dien-5-yl-compounds in Nothrus versus p-mentha-1,8-dien-3-yl-compounds in Astigmata

**Fig. 5:**
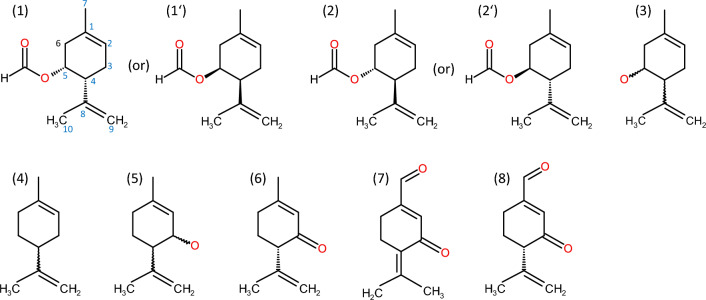
*p*-Menthanes in the oil glands of Oribatida and Astigmata. (1–3) *p*-Menthanes and substitution patterns in the secretion of adult *Nothrus palustris*: *p*-1,8-menthadien-5-yl-compounds. *p*-1,8-Menthadien-5-yl formates (*1, 2*) are the major *p*-menthanes in *N. palustris*, showing a formyl group in position 5 (following the *p*-1,8-menthadien-nomenclature). By NMR, the absolute stereochemistry of the formates (*1* or *1’*; *2* or *2’*) remained open. *p*-1,8-Menthadien-5-ol (*3*) was tentatively identified on the basis of its mass spectrum and proposed to represent the corresponding alcohol to the *p*-1,8-menthadien-5-yl formates. All compounds rely on a *p*-1,8-menthadien-5-yl-structure. 4–8) *p*-Menthanes and derivatives in the Astigmata: *p*-1,8-menthadien-3-yl-compounds. Limonene* (*p*-1,8-menthadiene), isopiperitenol (*p*-1,8-menthadien-3-ol), (*S*)-isopiperitenone (*p*-1,8-menthadien-3-one) (4), robinal, and isorobinal have been reported as astigmatid oil gland constituents (see Kuwahara 2004 and text). Apart from limonene, all compounds rely on a *p*-1,8-menthadien-3-yl-structure. *Limonene is the backbone-structure for both, *p*-1,8-menthadien-3-yl- and *p*-1,8-menthadien-5-yl-compounds

Menthane monoterpenoids represent a chemical class new to the oil gland secretions of Oribatida. The menthanes found in *N. palustris* contain one ring and two or three double bonds, and are based on/derived from a limonene backbone (= 4-isopropenyl-1-methyl-cyclohexene = *p*-mentha-1,8-diene). Oxygen-containing substituents, if present, preferably occur in position 5, suggesting *p*-mentha-1,8-dien-5-ols as leading structures (Fig. 5). Intriguingly, from the oil glands of Astigmata, several similar *p*-menthane monoterpenoids have been reported: Isopiperitenone, a *p*-mentha-1,8-dien-3-one, was found in the oil glands of species of *Tyrophagus* (Oudemans 1924a), *Tyroborus* (Oudemans 1924b), and *Schwiebea* Oudemans 1916. Further *p*-menthane derivatives, such as robinal and isorobinal, were described from *Rhizoglyphus*, *Tyroborus*, and *Schwiebea* (Kuwahara et al. 1987; Leal et al. 1990; Tarui et al. 2002; Tomita et al. 2003; Mizoguchi et al. 2005; Maruno et al. 2012). Kuwahara (2004) reviewed astigmatid oil gland constituents and additionally listed limonene (*p*-mentha-1,8-diene) and isopiperitenol (*p*-mentha-1,8-dien-3-ol). All these compounds from astigmatid mite oil glands share the *p*-mentha-1,8-diene-backbone with *Nothrus*, but differ in the position of the oxygen-containing moiety attached to the ring (which is in position 3 in Astigmata). Thus, oxygenated menthanes in Astigmata represent *p*-mentha-1,8-dien-3-ols and derivatives of these (Fig. 5).

The major components in the secretion of *N. palustris* were identified as two isomeric *p*-1,8-menthadien-5-yl formates. These findings shed new light on the initial study on *Nothrus*: Shimano et al. (2002) did not mention a *p*-menthane predominated secretion but show a figure (Fig. 1/4 in Shimano et al. 2002) of an adult chromatogram with a few unidentified peaks in characteristic positions (apart from dehydrocineole and heneicosadiene). We tentatively assume that these peaks, with much lower abundance in the Japanese population, might correspond to the herein described *p*-menthanes.

### p-Menthane subclasses in Nothrus

The *Nothrus*-menthanes may be classed into three groups, according to their molecular mass and degree of oxygenation, respectively. There are (i) tentatively identified *p*-menthane-hydrocarbon monoterpenes, (ii) such with one oxygen atom, and (iii) compounds with two oxygens. Diagnostic ions were given by the fragment *m/z* 134, which is a pure hydrocarbon fragment (C_10_H_14_^+^); whereas the fragment at *m/z* 137 contained one oxygen (C_9_H_13_O^+^). Thus, molecular ions at *m/z* 134, as present in compounds I and II, probably indicated hydrocarbon *p*-menthanes. For compounds I and II, there are assumed to be menthatrienes, but reported retention indices for several menthatrienes (e.g., Adams et al. 2012: *p*-1,3,8-menthatriene; *p*-1,5,8-menthatriene) did not fit to our compounds. For *p*-1,4,8-menthatriene, we found a reference spectrum, but no retention index (Thomas and Bucher 1970). Since all the prominent ions in the spectra of compounds I and II (at *m/z* 134, 119, and 91) are rather unspecific hydrocarbon ions, arising from M^+^ (C_10_H_14_^+^), M-CH_3_ (C_9_H_11_^+^), and M-C_3_H_7_ (C_7_H_7_^+^), this pattern is also found in the other components, such as cymols and in oxygenated compounds as carenol.

On the other hand, menthanes with M^+^ at *m/z* 152, as in compound III, generally possess one oxygen, and likely represent *p*-menthadienols of formula C_10_H_16_O. Our assumption for the compound III was *p*-mentha-1,8-dien-5-ol (= 6-isopropenyl-3-methyl-cyclohex-3-en-1-ol), for which no viable references are available. This molecule consists of a limonene backbone with a hydroxy group in position 5, analogously to the formates B and C. We, nevertheless, compared published spectra and retention indices for various other *p*-1,8-menthadienols to our compound III, not finding correspondence. Reference data for *p*-1,8-menthadienols were from the NIST webbook (including *p*-1,8-menthadien-4-ol; *p*-1,8-menthadien-6-ol = carveol; *p*-1,8-menthadien-7-ol; *p*-menthadien-9-ol) as well as from literature sources (e.g., cis- and trans carveol: Garneau et al. 1997; *p*-1,8-menthadien-3-ol = isopiperitenol: Lücker et al. 2004). Generally, *p*-1,8-menthadien-5-ol appears to be a rare compound in nature: we only found one old report on its occurrence, namely in the oil of a particular population of lemongrass *Cymbobogon flexuosus* (Naves 1961). A rudimentary mass spectrum for *trans*- and *cis*-*p*-1,8-menthandien-5-ol (showing the three most prominent ions only and not sufficient for a comparison) is given by Brenna et al. (2006).

The remaining menthane compounds in *N. palustris*, i.e., those with M^+^ at *m/z* 180 (compounds IV and V), may contain one or two oxygen atoms. Regarding the fragmentation of the *p*-mentha-1,8-dien-5-yl formates B and C, the neutral loss of formic acid from the molecular ion at *m/z* 180 leads to fragment *m/z* 134 (M^+^—HCOOH) and indicates two oxygens present in the molecule. Compounds IV and V showed spectra very similar to compounds B and C, possibly—but not indicatively—representing formates as well but with the formyl group in a different position. Their amounts were too low for a final identification by NMR. Compounds IV and V again exhibited intense ions at *m/z* 134 (C_10_H_14_^+^), but additional ions at *m/z* 137 (C_9_H_13_O^+^) as well. A loss of CH_2_COH from a molecular ion at *m/z* 180 (M-43) theoretically leads to *m/z* 137 (indicating two oxygens in the original molecule), whereas a loss of C_3_H_7_ would lead to *m/z* 137 as well (indicating one oxygen in the original molecule).

### Menthane functions, occurrence, and biosynthesis

Generally, *p*-menthanes rarely predominate in arthropod exocrine secretions, but some compounds of this group such as limonene, terpinene, and carvone are found in several insects, arachnids, and some millipedes (e.g., Blum 1981). While mostly acting as repellents, some *p*-menthanes in the secretions of Astigmata show pheromonal properties (Kuwahara et al. 1987; Mizoguchi et al. 2005). The biological functions of menthanes in *Nothrus* remain unstudied for the time being. On the other hand, *p*-menthanes in plants are frequent and abundant, characterizing the oils of plants of different families, such as Lamiaceae, Apiaceae, Rutaceae, and Eucalyptae (e.g., Lange 2015; Bergmann and Phillips 2021).

Geranial, the major component in the juvenile stages of *N. palustris*, is considered to arise via the mevalonate pathway that is well described for terpene-producing Oribatida (Brückner et al. 2022). In this pathway, geranyl pyrophosphate (GPP) represents a central intermediate from which various terpenes can be built. Regarding geranial, GPP has to be hydrolyzed into geraniol and pyrophosphate, and geranial may be produced by enzymatic oxidation of geraniol.

In case of menthanes, it is generally limonene that is produced from GPP by limonene synthetases (e.g., Bergmann and Phillips 2021). For oxygenated *p*-menthanes, a hydroxy group is introduced into limonene, and subsequently, *p*-menthadienols and also *p*-menthadienyl formates can be built. Schempp et al. (2021), for instance, describe a limonene-5-hydroxylase that adds a hydroxy group to (*S*)-limonene, thus leading to the formation of *trans*-*p*-1,8-menthadien-5-ol. In adult *N. palustris*, *p*-menthane monoterpenoids most likely also originate from the mevalonate pathway. Depending on the expression of appropriate sets of enzymes in juveniles and adults, we assume a split of the biosynthetic route at the level of geranyl pyrophosphate, which leads to geranial in juveniles but to *p*-menthanes in adults, and finally to juvenile–adult polymorphism of oil gland secretions in this particular species. We here call the phenomenon of changes in the composition of an exocrine secretion during ontogeny “chemo-metamorphosis”.

### Juvenile–adult polymorphism: chemo-metamorphosis

Geranial is a monoterpene compound frequently found in the oil glands of middle-derivative Oribatida and Astigmata. Besides neral, neryl formate, γ-acaridial, and 2,6-HMBD, it constitutes one of the so-called “astigmatid compounds” (e.g., Sakata and Norton 2001; Raspotnig 2010) that are characteristic of the oil gland secretions of middle-derivative Oribatida and Astigmata. No chemo-metamorphosis has been described for these groups, with juveniles basically producing the same secretions as adults (see introduction). Some derivative mixonomatans, such as some Euphthiracaroidea, already show an expanded, partly renewed compound-repertoire, including various iridoid monoterpenes. Interestingly, also these newly added compounds equally occur in both juveniles and adults (Raspotnig et al. 2008).

For some Desmonomata, however, and especially for late-derivative oribatid groups such as Brachypylina, an increasing number of examples for chemo-metamorphosis has been reported. *N. palustris* is only one of these examples, and possibly one of the rather early derivative examples of chemo-metamorphosis in the Oribatida. According to the oribatid catalog of Subias (2004; updated 2022), the Nothridae comprises about 100 species in three genera, with 87 species described for *Nothrus*. Notably, our preliminary data indicate that chemo-metamorphosis does not affect all species of genus *Nothrus* neither all Nothridae: initial analyses of the oil glands of several other Austrian species of *Nothrus* show a widespread production of geranial irrespective of ontogenetic state, i.e., including adults (Raspotnig, unpublished). This makes a generalized phenomenon of chemo-metamorphosis in the Nothridae unlikely. Chemo-metamorphosis also occurs in the Hermanniidae and later on in a number of late-derivative Brachypylina. In *Hermannia convexa* (C.L. Koch 1839), for instance, chemo-metamorphosis is present in an extreme form, even affecting the morphology of oil glands: juveniles produce astigmatid compounds from large oil glands, whereas the glands of adults become inactive and degenerate (Raspotnig et al. 2005a). The currently most striking example for chemo-metamorphosis may be found in species of *Scheloribates *(Berlese 1908) (Brachypylina, i.e., late-derivative Oribatida) which produce geranial as juveniles, but alkaloids as adults (Takada et al. 2005). In terms of Ernst Haeckel’s biogenetic law and his theory of recapitulation, respectively (Haeckel 1866: “ontogeny recapitulates phylogeny”), *Scheloribates*-juveniles might be considered to express phylogenetically ancient oil gland secretion characters which are replaced by a completely novel chemistry in adults. A comparable situation of chemo-metamorphosis may be true for many or even all alkaloid-producing taxa, thus for many Oripodoidea (e.g., Saporito et al. 2015).

Generally, the phenomenon of juvenile–adult polymorphism of exocrine secretions is not rare in arthropods, but currently available data are biased: irrespective of taxonomic group, mostly adult individuals have been investigated, and the ontogeny of secretion chemistry is only known for a minority of species. Chemo-metamorphosis, however, is known to occur in a number of insects (e.g., in bugs, see Aldrich 1988 for an overview), some polydesmidan millipedes (Kuwahara et al. 2015, 2019), exceptionally in Julida (Bodner and Raspotnig 2012), as well as in a few arachnids. In harvestmen, for instance, a first instance of juvenile–adult polymorphism of defensive secretions has recently been published (Raspotnig et al. 2022). Up to now, only a few species of late-derivative oribatid groups have been chemically investigated, and apart from *Scheloribates, Hermannia* Nicolet 1855, and *Nothrus*, investigations exclusively included adult individuals (e.g., Liacaridae: Raspotnig and Leis 2009; Brückner et al. 2015; Oripodoidea: Saporito et al. 2015; Brückner et al. 2017; Euphthiracaridae: Heethoff et al. 2018). We here hypothesize that chemo-metamorphosis is an adaptive trait in Oribatida that evolved somewhen in derivative Desmonomata, possibly to accommodate different ecological requirements of adults.
